# The effects of intramuscular tenotomy on the lengthening characteristics of tibialis posterior: high versus low intramuscular tenotomy

**DOI:** 10.1007/s11832-011-0335-5

**Published:** 2011-03-25

**Authors:** Altay O. Altuntas, Benjamin Dagge, Terence Y. P. Chin, Joseph E. A. Palamara, Norman Eizenberg, Rory Wolfe, H. Kerr Graham

**Affiliations:** 1 Orthopaedic Department, The Royal Children’s Hospital, Flemington Road, Parkville, VIC 3052 Australia; 2 Murdoch Childrens Research Institute, Flemington Road, Parkville, VIC 3052 Australia; 3 The University of Melbourne, Grattan Street, Parkville, VIC 3010 Australia; 4 Department of Anatomy and Developmental Biology, Monash University, Wellington Road, Clayton, VIC 3800 Australia; 5 Monash University, Commercial Road, Melbourne, VIC 3004 Australia

**Keywords:** Contracture, Lengthening, Muscle–tendon-unit, Tibialis posterior, Intramuscular tenotomy

## Abstract

**Background:**

Lengthening of soft-tissue contractures is frequently required in children with a wide variety of congenital and acquired deformities. However, little is known about the biomechanics of surgical procedures which are commonly used in contracture surgery, or if variations in technique may have a bearing on surgical outcomes. We investigated the hypothesis that the site of intramuscular tenotomy (IMT) within the muscle–tendon-unit (MTU) of the tibialis posterior (TP) would affect the lengthening characteristics.

**Methods:**

We performed a randomized trial on paired cadaver tibialis posterior muscle–tendon-units (TP-MTUs). By random allocation, one of each pair of formalin-preserved TP-MTUs received a high IMT, and the other a low IMT. These were individually tensile-tested with an Instron^®^ machine under controlled conditions. A graph of load (Newtons) versus displacement (millimetres) was generated for each pair of tests. The differences in lengthening and load at failure for each pair of TP-MTUs were noted and compared using paired *t* tests.

**Results:**

We found 48% greater lengthening for low IMT compared to high IMT for a given load (*P* = 0.004, two tailed *t* test). Load at failure was also significantly lower for the low IMT. These findings confirm our hypothesis that the site of the tenotomy affects the amount of lengthening achieved. This may contribute to the reported variability in clinical outcome.

**Conclusions:**

Understanding the relationship between tenotomy site and lengthening may allow surgeons to vary the site of the tenotomy in order to achieve pre-determined surgical goals. It may be possible to control the surgical “dose” by altering the position of the intramuscular tenotomy within the muscle–tendon-unit.

## Introduction

Considerable variation occurs in the location of the skin incision for intramuscular lengthening of tibialis posterior, in children with varus foot deformities, secondary to cerebral palsy and other conditions. Many factors may contribute to the unpredictability of outcome after muscle–tendon surgery, including the heterogeneity of the patient population under study [1, 2]. However, simple surgical issues such as the position of the incision for the IMT may also contribute.

Shortening of muscle–tendon-units (MTUs) can result in restriction of joint range of motion and impaired function [3]. MTU shortening due to contractures is a common feature of many congenital and acquired deformities in orthopaedic practice, especially neuromuscular disorders [4, 5]. Muscle contractures have been identified as a key component of the progressive musculoskeletal pathology which is characteristic of cerebral palsy [6]. Contracted MTUs may be lengthened by a variety of techniques, including simple tenotomy, *z*-lengthening, fascial striping, proximal or distal recession and intramuscular tenotomy (IMT). The choice may, in part, be determined by the anatomy of the specific MTU. Although the lengthening characteristics of intact MTUs have been extensively studied and well-reported [4, 5, 7–10], there is less information regarding simulated surgical procedures. It is also known that the outcome of surgical tendon lengthening procedures can be highly variable, especially in neuromuscular diseases such as cerebral palsy [1, 3–6, 11–16]. In recent years, there has been an emphasis on more conservative muscle–tendon surgery in such diverse fields as cerebral palsy and adult stroke, with the aim of preserving function and strength [4, 10]. Little information exists to guide surgeons in respect of the choice of lengthening procedure, or the correct level for IMT. Clinical observation suggests that the placement of incisions over MTUs is highly variable, suggesting that the choice of site for IMT is not well-defined. In turn, this may contribute to variability in surgical outcomes.

A range of surgical procedures on the tibialis posterior have been reported for the management of the varus hindfoot [2, 3, 11–13, 16–23], including complete tenotomy [22], *z*-lengthening of the tendon, intramuscular tenotomy [17, 19], re-routing the tendon anterior to the medial malleolus [13, 18, 24], split posterior tibial tendon transfer [20, 21], anterior interosseus transfer [13, 16, 24, 25], and combinations thereof [12, 26]. However, it is also recognized that the outcome of surgery for the varus hindfoot in cerebral palsy is extremely variable, with high rates of both recurrent deformity and over-correction [2, 3, 12, 14–16, 20, 26–28]. This may be related to heterogeneity within the patient population: however, it may also be due to variability in the execution of certain surgical procedures, including intramuscular tenotomy of tibialis posterior. Simple tenotomy and anterior interosseous transfer have unpredictable outcomes, have been condemned and largely discarded [2, 16, 22, 28]. Intramuscular lengthening of the tibialis posterior is a popular procedure in the management of the varus foot in spastic cerebral palsy, and is considered to have more predictable results [1, 14, 28].

**Fig. 1:**
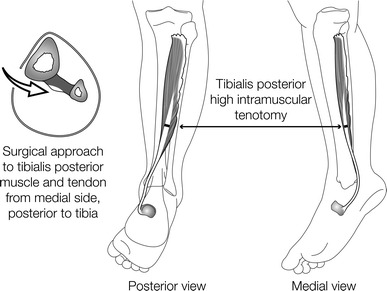
Equinovarus right foot and contracted TP-MTU with IMT, before stretching. Tibialis posterior tendon fibres divided (with *arrows*). Surgical approach to TP-MTU from medial side, posterior to tibia. Posterior view, medial view

**Fig. 2:**
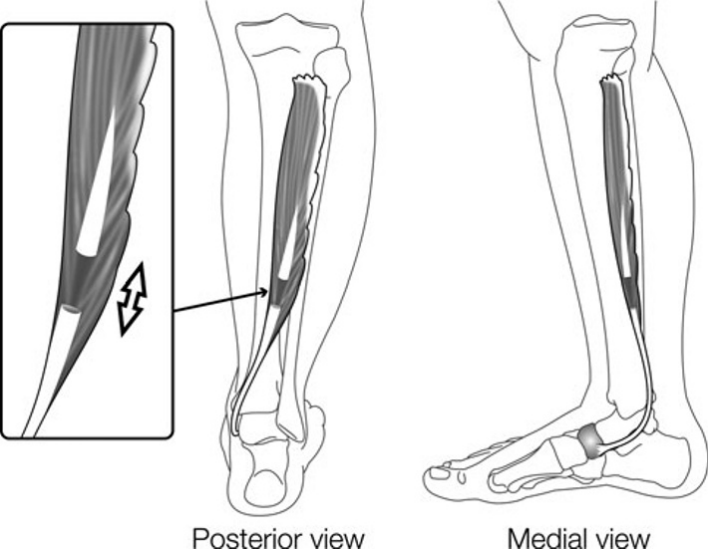
Right leg with tibialis posterior post Majestro, Ruda & Frost lengthening. Posterior view, medial view

The technique of intramuscular tenotomy of the tibialis posterior was first described by Maestro et al. in 1971 [17]. This technique is shown schematically in Figs. 1 and 2. Since then, the procedure has been reported to give reasonably consistent results, especially in children with mild deformities [1, 12, 14, 27, 28]. However, both in the original and subsequent descriptions of the procedure, the level of the IMT was poorly described. We investigated the effects of altering the position of the IMT within the substance of the tibialis posterior, in the adult formalin-preserved TP-MTUs. We wished to know if the amount of lengthening could be altered by changing the location of the IMT, within the belly of the tibialis posterior.

## Materials and methods

The regulations controlling the handling of cadaveric tissue were followed (Human Tissue Act of Victoria, Australia 1982). Twenty TP-MTUs were dissected from ten formalin-preserved human cadavers. Mean age was 82.0 years (range 50–94 years), with five male and five female cadavers. The cadavers were fixed using 2% formaldehyde, 2.7% phenol, 33% glycerin and water. Formalin fixation does not change the mineral composition or structure of tissue, but it can cause stiffness [29]. Examination of the cadavers demonstrated stiffness of all major joints. The ankles and feet were in equinovarus, the resting posture post mortem, fixed during the formalin preservation. In the posterior compartment of the leg, the fleshy origin of the tibialis posterior was sharply dissected to free it from the fibula, tibia and interosseous membrane. On the medial side of the foot, the tendon of insertion was also sharply dissected to free it particularly from the navicular and medial cuneiform.

**Fig. 3:**
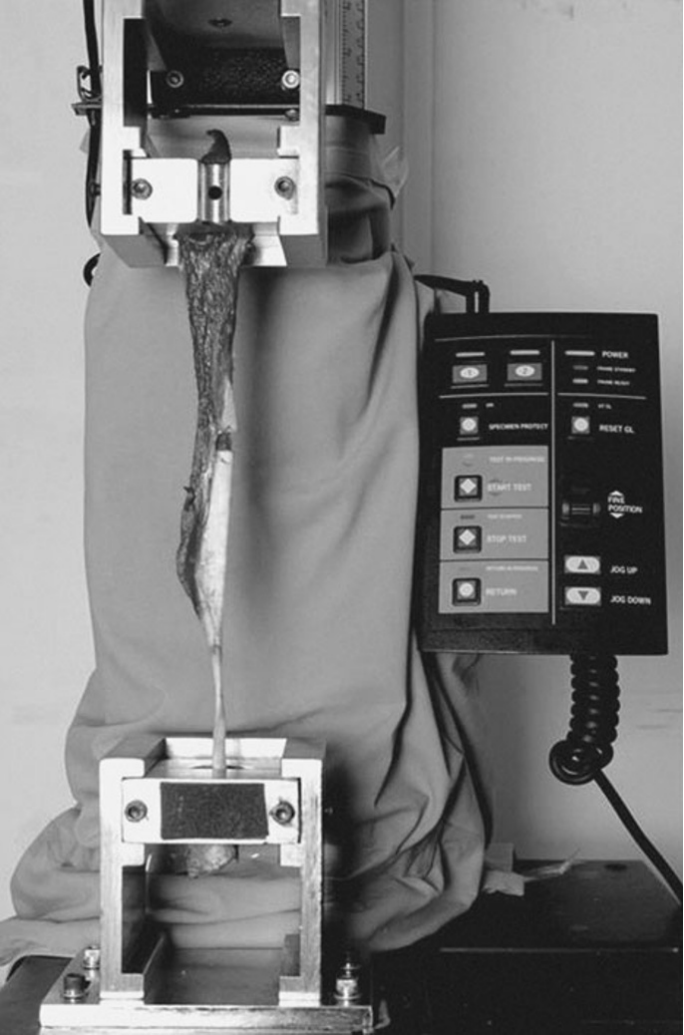
Experimental set-up: TP-MTU with high IMT undergoing tensile testing using Instron^®^ Model 5544 testing machine

Using an Instron^®^ Model 5544 (Instron Corporation, Canton, MA, USA) electromechanical testing machine operated with Merlin II Materials Testing Software (M12-15500, 1996) tensile testing of cadaveric TP-MTUs was conducted according to the following protocol. The TP-MTUs were mounted using specially manufactured metal clamps to minimize compliance in the system. A preload of 1 Newton (N) was initially placed on the specimen to ensure correct orientation for loading. Compliance of the system (elongation of clamps and rig) was tested, and found to be insignificant relative to the muscle lengthening. The experimental set-up is shown in Fig. 3.

A series of paired, numbered opaque envelopes were prepared with each pair containing the instruction “high tenotomy” or “low tenotomy”. Once the TP-MTU was mounted and ready for testing, one of the next pair of envelopes was opened, and the appropriate tenotomy performed.

**Fig. 4:**
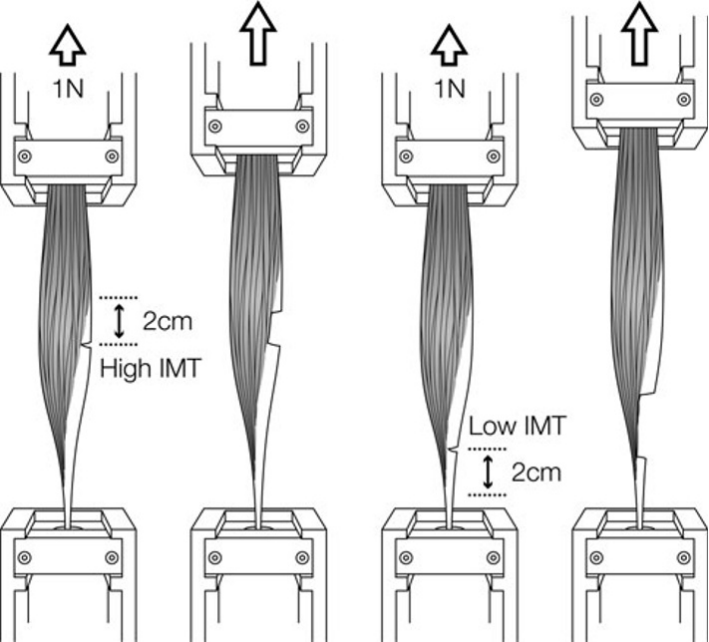
Schematic representation of TP-MTU high and low IMT tensile testing

An intact tibialis posterior was mounted and tested to failure without tenotomy as a control. The corresponding TP-MTU was randomized to low tenotomy but excluded from the statistical analysis to allow paired *t* tests. The remaining nine pairs of TP-MTUs provided the trial material. Once mounted, a transverse IMT was applied using a number 15 scalpel at either a high or low position within the MTU. *Low* tenotomy was performed 2 cm proximal to the *termination* of the distal muscle fibres. *High* tenotomy was performed 2 cm from the *commencement* of the tendon at the proximal end of the MTU (Fig. 4).

**Fig. 5:**
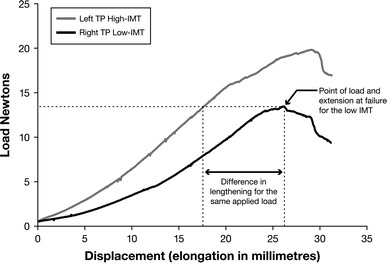
Results for high and low tibialis posterior intramuscular tenotomy from cadaver 8. (In *box*) Left TP High IMT, Right TP Low IMT (with *corresponding lines*), Point of load at failure for the paired low IMT (with *dashed lines*), Difference in lengthening for the same applied load (with *arrows*), (*X*-axis note units 0–25 N) Load (Newtons), (*Y*-axis, note units 0–35 mm) Displacement (elongation in millimetres), (Note also graph starts at “preloaded” 1 Newton on the *X*-axis)

Following tenotomy, each mounted TP-MTU was tensile tested to failure, at a rate of 5 mm per minute. This rate was based on experience with materials of similar characteristics. Force was recorded using a 2,000 N load cell with a resolution of 0.25 N. The load cell was calibrated before each experimental run. Load (N), displacement (elongation in millimeters) and time were recorded synchronously for each experiment. This data (load, displacement and time) was collected at a rate of two Hertz. Data was stored in Microsoft Excel^®^ (Microsoft Corporation, USA) compatible format for further statistical analysis (Table 1). Graphs of load versus displacement were generated for each pair of TP-MTUs (Fig. 5). The load and displacement at failure was determined for each pair of MTUs. Displacement after high IMT, at the same applied load as for low IMT failure, was also determined from the graphs. The mean of these values were compared using paired *t* tests.

**Table 1:** Results for tibialis posterior intramuscular tenotomy tensile testing

Cadaver	Side	Tenotomy	Load (N) at failure	Displacement (mm) at failure
1	Left	None (control)	157.1	10.0
1	Right	Low	9.2	6.3
2	Left	High	38.8	28.4
2	Right	Low	8.9	15.5
3	Left	Low	3.2	6.0
3	Right	High	11.1	15.3
4	Left	Low	4.4	6.8
4	Right	High	17.7	39.4
5	Left	Low	3.3	7.6
5	Right	High	10.3	16.3
6	Left	Low	4.3	7.6
6	Right	High	14.2	14.4
7	Left	High	35.8	35.4
7	Right	Low	13.8	16.0
8	Left	High	19.8	29.0
8	Right	Low	13.5	26.1
9	Left	High	35.5	34.4
9	Right	Low	28.3	35.4
10	Left	Low	7.0	20.3
10	Right	High	20.6	30.6

## Results

Figure 5 is a representative graph of the load versus displacement curves for high and low tenotomy, from one pair of MTUs (cadaver 8). The results for load at failure and displacement at failure for each MTU tested are found in Table 1. From this data, the following summary statistics and significance testing are derived.

The *control* intact tibialis posterior (cadaver 1, left) was subjected to a load of 157 N. The control lengthened by 10 mm *without* tenotomy prior to failure. By contrast, the contralateral MTU (cadaver 1, right) lengthened 6.3 mm with an applied load of 9.2 N following IMT. At the corresponding load of 9.2 N the intact TP-MTU had lengthened by 2.1 mm.

The *mean lengthening at failure*, following a low IMT was 15.7 mm (SD 10.1 mm, 95% CI 7.9–23.5 mm). The mean lengthening for a high IMT at the same applied load (matched to failure point for the paired tibialis posterior (TP) low IMT) was 10.6 mm (SD 7.8 mm, 95% CI 4.6–16.6 mm). The mean difference in length for high versus low IMT at same applied load were compared with paired *t* tests and found to be significant with a value of *P* = 0.004 (two tailed). A low IMT resulted in 48% more lengthening than a high IMT (15.7 mm vs. 10.6 mm).

The *mean load at failure* was significantly lower for the low intramuscular tenotomies compared to the high intramuscular tenotomies. The mean load at failure for low IMT was 9.6 N (SD 8.1, 95% CI 3.4–15.9 N) whilst mean load at failure for high IMT was 22.6 N (SD 11.2, 95% CI 14.1–31.2 N). This is more than double the load for low IMT. The mean difference in load at failure was 13.0 N. These results were compared using paired *t* tests and found to have a *P* value of 0.001 (two-tailed).

## Discussion

In this study, we investigated the effects of “high” versus “low” intramuscular lengthening of the human cadaver tibialis posterior tendon, as described by Majestro et al. [17]. In the original description, as well as more recent textbooks, there is a paucity of information as to where the IMT should be performed [1, 2, 17]. Additionally, the morphology of muscle–tendon-units in children with cerebral palsy differs significantly from that in subjects without neuromuscular disease. In cerebral palsy the muscle bellies tend to be shorter and the tendons longer in affected individuals [3, 5]. Hence the muscle–tendon junction may vary considerably according to the severity of involvement with more severe individuals having more severe contractures, shorter muscle bellies and longer tendons [6].

In the intact, formalin preserved human cadaver, we found that the gastrocsoleus and tibialis posterior muscles were short and stiff and had caused equinovarus contractures of the foot and ankle. These contractures have *some* similarities to the equinovarus contractures found in chronic neuromuscular diseases. We suggest that the formalin preserved human cadaver MTU may be a reasonable model for biomechanical testing and useful proxy for the simulation of surgical procedures for the correction of fixed contractures. Formalin fixation does not change the mineral composition or structure of tissue but it can cause stiffness [29]. However, we recognize the limitations of the model and the dangers of extrapolating findings based on bench top testing to the clinical situation. The clinical situation is much more complex, because the effects of spasticity are superimposed on the effects of contracture. Despite the increasing use of dynamic assessments such as gait analysis, it is usual practice to perform an examination under anesthesia (EUA), prior to lengthening MTUs, for fixed contracture. At that moment, the surgeon must determine where to locate the IMT. We do not suggest that *quantitative* extrapolations can be made from our work to the situation at EUA. Rather we suggest that there is a simple underlying *principle*, which is that the site of the MTU determines the lengthening characteristics.

Intramuscular lengthening of MTUs is widely used to correct fixed contracture, with the aim of improving gait and function in many neuromuscular disorders including cerebral palsy and stroke. Intramuscular tenotomy is the preferred technique of many authors, in recent literature and standard texts [1, 2, 11, 12, 14, 17–19, 23, 28]. The postulated benefits of intramuscular lengthening are that it is a relatively simple technique; it may permit lengthening in continuity and preserve the function of the MTU. Despite the interest in conservative methods of muscle–tendon lengthening and many studies reporting the outcomes of such procedures, there have been few comparative studies, no randomized clinical trials and few biomechanical investigations in which the effects of muscle–tendon surgery have been simulated.

Following IMT, there is also limited information regarding the mechanism by which the MTU is manually lengthened and the stability of the lengthened unit. This leads to lack of consensus and uncertainty in respect of advice regarding weight bearing status. There are obvious limitations on the ability to investigate some of these issues in clinical studies, and for these reasons we performed this randomized trial in human cadavers. We standardized the procedure as an intramuscular tenotomy, within 2 cm of the *commencement* of the tendon or within 2 cm of the *termination* of the muscle fibres. This was a deliberate attempt to exaggerate differences that might be detected in the lengthening characteristics of the MTU, given the limited cadaveric resource.

The intact tibialis posterior (without IMT) was found to be very stable and resistant to lengthening, during loading (Table 1). An IMT reduced the resistance to lengthening by an order of magnitude, and allowed lengthening in continuity. The graphs of load versus lengthening were similar to those described for intact human and animal tendons, including a “toe” section, followed by a straight-line relationship during which Hooke’s law applies, followed by plastic deformation and failure [30, 31]. The results of the experiment were highly consistent in that lengthening was greater for the same applied load, and load at failure was lower for the low IMT compared to the high IMT. Given that lengthening in continuity occurs through the intact muscle fibres and supporting connective tissue, we think that the difference in lengthening characteristics reflects the difference in cross-sectional area of the remaining intact muscle at the level of the IMT. However, a weakness of our study was that cross-sectional area was not measured.

The implications of our findings are that predictable lengthening of muscle–tendon-units is possible following IMT and application of progressive loading. The amount of lengthening and the stability of the lengthened MTU are both affected by the position of the tenotomy within the MTU. The relationship between the loads applied in these experiments and those applied by surgeons intra-operatively as well as those experienced during rehabilitation, are unknown.

We readily concede that the clinical situation, in terms of surgical management of contracted MTUs in a child with CP, is an order of magnitude more complex than the isolated MTU harvested from an adult cadaver. We do not wish to imply that the *quantitative* data relates to the clinical situation but rather the *principle*. That is, we observed a highly consistent *trend* in the cadaver material showing that the site of the IMT affected the lengthening characteristics of the MTU. We suggest that this *principle* may apply to a degree in the clinical situation, but make no assumptions in respect of the quantitative aspects of our study.

The position of the tenotomy is under the control of the surgeon, and these findings may have direct implications for contracture surgery in such diseases as cerebral palsy and stroke. We suggest that surgical “dose” may be altered by changing the position of the intramuscular tenotomy within the muscle–tendon-unit. For severe or recurrent deformities, a larger surgical dose may be chosen, a distal intramuscular tenotomy performed, and greater lengthening and deformity correction may ensue. Conversely, a smaller “dose” may be required for minor degrees of deformity in some younger children, when a more proximal intramuscular tenotomy might be appropriate. These hypotheses will require careful investigation in clinical trials.
